# Investigations of the Microvasculature of the Human Macula Utricle in Meniere’s Disease

**DOI:** 10.3389/fncel.2019.00445

**Published:** 2019-10-04

**Authors:** Gail Ishiyama, Ivan A. Lopez, Dora Acuna, Akira Ishiyama

**Affiliations:** ^1^Department of Neurology, David Geffen School of Medicine at UCLA, Los Angeles, CA, United States; ^2^Department of Head and Neck Surgery, David Geffen School of Medicine at UCLA, Los Angeles, CA, United States

**Keywords:** microvasculature, macula utricle, pericytes, Meniere’s disease, blood labyrinthine barrier, vascular endothelial cells, glucose transporter-1

## Abstract

The integrity and permeability of the blood labyrinthine barrier (BLB) in the inner ear is important to maintain adequate blood supply, and to control the passage of fluids, molecules and ions. Identifying the cellular and structural components of the BLB, the vascular endothelial cells (VECs), pericytes, and the perivascular basement membrane, is critical to understand the pathophysiology of the inner ear microvasculature and to design efficient delivery of therapeutics across the BLB. A recent study of the normal and pathological ultrastructural changes in the human macula utricle microvasculature demonstrated that the VECs are damaged in Meniere’s disease (MD), and further studies identified oxidative stress markers (iNOS and nitrotyrosine) in the VECs. Using fluorescence microscopy, the microvasculature was studied in the macula utricle of patients diagnosed with MD that required transmastoid labyrinthectomy for intractable vertigo (*n* = 5), and patients who required a translabyrinthine approach for vestibular schwannoma (VS) resection (*n* = 3). Normal utricles (controls) were also included (*n* = 3). VECs were identified using rabbit polyclonal antibodies against the glucose transporter-1 (GLUT-1) and pericytes were identified using mouse monoclonal antibodies against alpha-smooth muscle actin (α-SMA). Immunofluorescence (IF) staining was made in half of the utricle and flat mounted. The other half was used to study the integrity of the BLB using transmission electron microscopy (TEM). GLUT-1-IF, allowed delineation of the macula utricle microvasculature (located in the stroma underneath the sensory epithelia) in both MD and VS specimens. Three sizes of vessels were present in the utricle vasculature: Small size (<15 μm), medium size (15–25 μm) and large size >25 μm. α-SMA-IF was present in pericytes that surround the VECS in medium and thick size vessels. Thin size vessels showed almost no α-SMA-IF. AngioTool software was used for quantitative analysis. A significant decreased number of junctions, total vessel length, and average vessel length was detected in the microvasculature in MD specimens compared with VS and control specimens. The deeper understanding of the anatomy of the BLB in the human vestibular periphery and its pathological changes in disease will enable the development of non-invasive delivery strategy for the treatment of hearing and balance disorders.

## Introduction

The inner ear cochlear and vestibular function is dependent on the integrity of the vasculature for the maintenance of an adequate blood supply, control of metabolism, and fluid homeostasis (Shi, 2016). The blood labyrinthine barrier (BLB) plays a critical role in the ionic transport needed to maintain the highly differing ionic composition of the endolymph and the perilymph, creating the electrophysiological gradient necessary for hearing and balance function. Recent studies in the blood brain barrier (BBB) demonstrate that several environmental factors, mental stress, noise exposure, air pollution affects vascular endothelial function (Daiber et al., 2017), and a similar pathophysiology may occur in the inner ear vasculature in response to environmental insults.

The anatomy of the human inner ear vasculature has been recently described (Mei et al., 2018) and a detailed anatomical review was given by Mudry and Tange (2009). In their review, they confirmed the spatial organization of the inner ear vasculature illustrated by Nabeya in 1923 (Nabeya, 1923). Mazzoni describes in detail the vascular anatomy of the normal vestibular labyrinth in humans using 1 mm thick sections of decalcified temporal bones stained with osmium tetroxide and cleared with methylsalycylate Mazzoni (1990).

In the last 20 years considerable advances have been made to understand the physiology and cellular, molecular structural organization of the BLB in animal models (Shi, 2016). The BLB in the rodent model is a complex structure formed by VECs, pericytes, and the underlying perivascular basement membrane (Shi et al., 2008; Neng et al., 2013a, b; Zhang et al., 2013). The first detailed anatomical and ultrastructural study of the human vestibular BLB demonstrated similarities of the human BBB and the animal BLB (Ishiyama et al., 2017).

Our recent studies have demonstrated pathology of the BLB in MD (Ishiyama et al., 2017, 2018), a disabling inner ear disorder characterized by incapacitating attacks of vertigo, hearing loss, and tinnitus (Baloh, 2001; Ishiyama et al., 2001). Histopathological studies have demonstrated a nearly universally associated hydrops of the endolymphatic system of the inner ear, a ballooning of the endolymph structures (Hallpike and Cairns, 1938; Yamakawa, 1938; Schuknecht, 1993). However, the role of hydrops in MD remains unknown (Merchant et al., 2005; Semaan et al., 2005; Semaan and Megerian, 2010; Foster and Breeze, 2013; Ishiyama et al., 2015). While it has been proposed that hydrops is an epiphenomenon (Merchant et al., 2005), we believe that given the near universality of hydrops in cases of MD, that hydrops is a necessary but not sufficient factor for MD. The lack of an animal model for MD that fully exhibits the clinical features hampers the identification of the cellular and molecular pathophysiological mechanisms.

Several studies using MRI with gadolinium in patients diagnosed with MD have demonstrated endolymphatic hydrops of the affected inner ear of MD subjects (Ishiyama et al., 2015; Sepahdari et al., 2015, 2016; Pakdaman et al., 2016). In these studies which utilize gadolinium enhanced MRI protocols to image endolymphatic hydrops, patients with MD exhibit significantly greater contrast enhancement in the affected ear perilymph (Tagaya et al., 2011; Yamazaki et al., 2012; Barath et al., 2014; Pakdaman et al., 2016). Intravenous gadolinium is taken up into the perilymph, presumably via perfusion through the BLB, specifically the blood-perilymph barrier. In animal studies, substances that break down the BLB when introduced intratympanically, such as lipopolysaccharide, cause ipsilateral increased perilymph (LeFloc’h et al., 2014) or increased entry of serum fluorescein into the perilymph (Hirose et al., 2014), demonstrating that an increase in BLB permeability can cause ipsilateral increased perilymph gadolinium signal on MRI.

Histopathological studies of the morphological alterations of the inner ear vasculature from MD patients have revealed basement membrane pathology in the BLB (McCall et al., 2009). Further evaluations demonstrated epithelial and perivascular basement membrane constituent protein alterations (Calzada et al., 2012), suggesting that the pericytes of the BLB may be affected in MD. The pericytes surround the abluminal face of the VECs tube and extend long cellular processes along the abluminal face of the VECs. In the rodent BLB, pericytes contain contractile proteins and have the ability to contract to control the diameter of the capillary (Shi et al., 2008). To the date there are not specific markers for pericytes, however, several antibodies have been used to identify them: α-smooth muscle actin (α-SMA), platelet-derived growth factor receptor-β (PDGFRβ), NG2, desmin and other markers (Shi et al., 2008). The ultrastructure of blood vessels located in the stroma of the MD human macula utricle has demonstrated increased vesicle formation and degeneration of the VECs of the BLB (Ishiyama et al., 2017). The expression of two oxidative stress markers: inducible nitric oxide synthase (iNOS) and nitrotyrosine in VECs of the BLB of the human vestibular utricle from Meniere’s patients implicates oxidative stress as mediating the disruption of the BLB (Ishiyama et al., 2018).

In the present study we hypothesize that the vasculature of the BLB of the macula utricle will exhibit structural alterations and constrictions in MD, and that the cellular components of the BLB will exhibit degenerative changes. There is evidence that leakage of the microvasculature of the inner ear may result in edema triggering MD symptoms (Ishiyama et al., 2015). Recent progress on BLB pathophysiology in animal models highlights the importance of the BLB integrity for ion homeostasis, prompting us to question whether dysfunction of the BLB is key to understand the pathophysiology of MD (Shi, 2011, 2016; Hirose et al., 2014). We detected increased transcellular vesicular transport across VECs, detachment of pericyte processes, and disruption of perivascular basement membrane in the stroma beneath the macula and cristae vestibular sensory epithelia in MD patients (Ishiyama et al., 2017, 2018).

We have examined and described in detail the histopathology of vestibular endorgans (macula and cristae) obtained from MD patients (McCall et al., 2009; Ishiyama et al., 2017, 2018). In the prior histopathological studies of the BLB microvasculature in MD, we noted a narrowing of the lumen which may affect the perfusion of the auditory and vestibular neuroepithelium and nerve (Ishiyama et al., 2017, 2018). However, these observations were made in thin cross sections, making it difficult to identify the extent of alterations in the vasculature in the whole endorgan. Whole mount immunofluorescence staining and quantification of changes in the vascular network using AngioTool software (Zudaire et al., 2011) was made in the macula utricle obtained by ablative surgery from MD and vestibular schwannoma (VS) patients and normal (control) utricles obtained from microdissected temporal bones obtained at autopsy. The specimens were immunostained with antibodies against glucose transporter-1 (GLUT-1) to visualize VECs and α-SMA to identify pericytes in the utricle microvasculature.

## Materials and Methods

### Sources of Specimens

Appropriate informed consent for inclusion in the study was obtained from each donor. Approval was obtained from the University of California at Los Angeles Institutional Review Board (IRB protocol # 10-001449). Vestibular endorgans were acquired at surgery from patients diagnosed with MD that required transmastoid labyrinthectomy for intractable vertigo (*n* = 5, 2 male: 43 and 58 years old (specimen #1 and #2; female: 52, 61 and 69 years old: specimens #3, #4 and #5), patients who required a translabyrinthine approach for VS resection (*n* = 3, male: 60 years old, specimen # 6; female: 53 and 58 years old: specimens #7 and #8). Normal utricles microdissected from temporal bones obtained at autopsy (2 females age 68, 70 years old, specimen #9 and #10 respectively, and 1 male 75 years old, specimen #11) were also included in this study (Table 1).

**TABLE 1:** Parameters computed by AngioTool.

**Specimen**	**Type**	**Vessels area**	**Vessels %**	**Number of**	**Total vessels**	**Average vessels**	**Total number of**
		**(mm^2^)**	**area**	**junctions**	**length (mm)**	**length (mm)**	**end points**
1	MD	0.064	20.13	46	5.32	0.10	139
2	MD	0.098	30.08	63	8.05	0.14	158
3	MD	0.043	15.35	18	3.84	0.10	87
4	MD	0.086	27.90	57	6.96	0.11	159
5	MD	0.11	35.92	92	9.70	0.08	106
6	VS	0.13	39.88	201	11.71	0.18	262
7	VS	0.11	34.78	119	11.16	0.13	313
8	VS	0.10	25.55	239	12.64	0.19	252
9	C	0.113	23.33	133	13.37	0.17	274
10	C	0.105	32.14	205	13.15	0.13	251
11	C	0.121	20.30	234	12.85	0.16	343

*MD, Meniere’s disease; VS, vestibular schwannoma; C, control. Total area analyzed 0.578 × 0.578 mm = 0.334 mm^2^.*

### Inclusion Criteria

The following criteria were required for surgical specimens diagnosed with MD: (1) Documentation that the patient meets the 1995 AAO-HNS criteria for definite MD. (2) Ipsilateral non-serviceable hearing. (3) Intractable to medical therapy including low salt diet and diuretics.

### Tissue Processing

**FIGURE 1:**
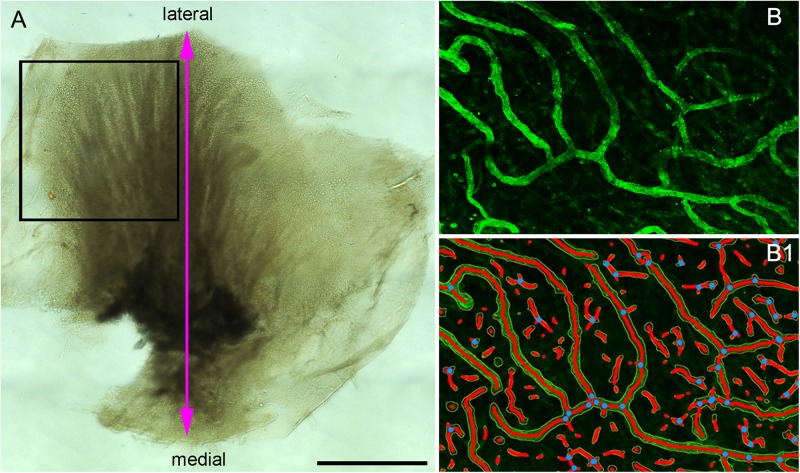
**(A)** The macula utricle was cut into two halves. The black color rectangular frame shows the area analyzed for quantitative immunoflurescence. **(B)** A small immunostained area of the macula utricle (Specimen on Figure 4C) and **(B1)** shows the resulting segmented and skeletonized image from **(B)**. Blue dots showed the branching index. Bar in **(A)** is 1 mm.

Utricles obtained from ablative surgery or microdissected from temporal bones were fixed in 10% formalin overnight, washed with phosphate buffered saline solution (PBS, 0.1M, pH 7.4) and cut in two halves under the dissecting microscope (Figure 1A). The cut was made from the medial (nerve stump) to the lateral portion. One half was used for immunofluorescence staining and one half was processed for transmission electron microscopy (TEM). Tissue used for immunofluorescence staining was immersed in 30% sucrose in PBS for 1 day for cryoprotection. The following day the tissue was snap frozen in liquid nitrogen, thawed (Lopez et al., 2005) and processed for immunofluorescence staining (Lopez et al., 2016).

### Immunofluorescence (Whole Mount Preparation)

Tissue was placed in a rotary shaker and incubated for 3 h in a blocking solution containing 2% bovine serum albumin fraction V (Sigma, SLM), 0.1% Triton X-100 (Sigma, SLM) diluted in PBS at 4–6°C. Subsequently, the blocking solution was removed and the half utricles are incubated for 72 h with the primary antibodies, placing the vials in the rotatory shaker in a cold room. At the end of the incubation, the blocking solution was removed and the primary antibodies against GLUT-1 (1:1000 in PBS) and α-SMA (1:1000 in PBS) were incubated 48 h at 4°C in a humidity chamber. GLUT-1 (Abcam, is a rabbit polyclonal antibody with specific reactivity for human and rat, raised against a synthetic peptide), α-SMA is a mouse monoclonal antibody (SIGMA-ALDRICH, specific reactivity for human, mouse and rat, generated with an N-terminal synthetic decapeptide of α-SMA). The secondary antibodies against rabbit or mouse labeled with Alexa 488 or 594 (1:1000, Molecular Probes, Carlsbad, CA, United States) were applied and incubated for 2 h at room temperature in the dark. At the end of the incubation, the whole endorgans or sections were washed with PBS (20 min × 5) and mounted flat on glass slides with Vectashield solution containing DAPI (Vector Labs, Burlingame, CA, United States) to visualize all cell nuclei. Specimens #3, #4, #5, and #8 were cut in two halves. One half was used for IF and the second half was used for TEM.

### Positive Controls

Cryostat sections were obtained from the macula utricle microdissected from temporal bones (contralateral side, specimens #9, #10, and #11 (IRB protocol # 10-001449). Sections were immunostained as described above. GLUT-1-IF delineate VECs and α-SMA-IF pericytes in the blood vessels located in the stroma utricle (same immunolocalization as shown in Ishiyama et al., 2018).

### Negative Controls

As negative controls, the primary antibody was omitted or preabsorbed with the antigen as described previously (Lopez et al., 2016; Ishiyama et al., 2018) and the immunoreaction was performed as described above. No immunoreaction was detected in both types of negative controls.

### Confocal Imaging

Fluorescent images were acquired using a high-resolution laser confocal microscope light-sheet (Leica, model SP8). For the region of interest (illustrated in new Figure 1A), series of approximately 50 digital GLUT-1-IF images (0.5 micron thick) were collected by the confocal microscope, and individual stacks were obtained. The first image was collected from the top of the stack where GLUT-1-IF blood vessels begin to appear (flat mount preparation) (generally below the sensory epithelia) until the bottom of the stack where the GLUT-1-IF blood vessels are no longer visible. Then a maximum projection image was created from each stack and this maximum projection image was used for quantification. All images were prepared using the Adobe Photoshop software program run in a Dell OptiPlex 3020 computer.

### Quantitative Analysis of the Vascular Network Using AngioTool Software

This software allows the quantification of vascular networks in microscopic immunofluorescence stained images and computes several morphological and spatial parameters: area covered by the vasculature vascular network, the number of vessels, vessels length, vascular density and lacunarity, and branching index. Confocal images of immunostained utricles with GLUT-1 were analyzed using this program (Open source)^1^ and processed as described (Zudaire et al., 2011). Images for quantitative analysis were acquired with the confocal microscope using a 20× objective (oil) at 1 zoom (200×). The image is immediately segmented, skeletonized and the analysis of the vasculature is performed automatically, the resulting image shows the overlay of the area of all vessels and shows also the computed branching points inside the area imaged. Figure 1A indicates the region of interest (ROI) analyzed (black color frame to the left side of the utricle). Figure 1B, illustrates the blood vessels immunostained with antibodies against GLUT-1. Figure 1B1 shows the segmented and skeletonized (delineated) blood vessels from which quantitative analysis was made. Branching points are seen in blue color. The image generated by the software was saved as TIFF file and an Excel file containing the computed results was generated.

Quantification was made double blinded, i.e., the researcher performing the immunostaining and the quantification was only given the specimen number (Table 1). At the end of the analysis each specimen was identified as MD, VS or control. The area examined per specimen (image field) used was 0.578 × 0.578 mm = 0.335 mm^2^. The GLUT-1-IF green color (channel) was selected because of the consistent delineation of the blood vessels (VECs). The following parameters were quantified: vessels area (the area of the segmented vessels), vessel percentage area (the percentage of area occupied by vessels inside the area examined), total number of junctions (the total number by vessels inside the explant area), total vessel length (the sum of Euclidean distances between pixels of all the vessels in the image), average vessel length (the mean length of all the vessels in the image and the total number of endpoints (the number of open ended segments).

### Statistics

Comparisons of the parameters described above were made as follow: Meniere’s vs. VS, MD vs. controls and VS vs. controls (Tables 2A–C). A student *t-*test for two independent means was obtained using the IBM SPSS statistics software program version 25 (IBM Corporation, Armonk, NY, United States). A value of *p* < 0.05 was denoted as a statistically significant difference.

**TABLE 2A:** Statistical comparisons MD vs. VS (*t*-test for 2 independent means).

**Parameter**	***t*-value**	***p*-value**	**Statistical significance**
			**at *p* < 0.05**

Vessels area	−1.91966	0.051658	No
Vessels% area	−1.30905	0.119207	No
Number of junctions	−4.31097	0.002516	Yes
Total vessels length	−3.61488	0.005583	Yes
Average vessels length	−3.22271	0.009038	Yes
Total number of end points	−6.178	0.000413	Yes
			

**TABLE 2B:** Statistical comparisons MD vs. Control (*t*-test for 2 independent means).

**Parameter**	***t*-value**	***p*-value**	**Statistical significance**
			**at *p* < 0.05**

Vessels area	−2.00431	0.045939	Yes
Vessels% area	0.11231	0.457121	No
Number of junctions	−4.98871	0.00124	Yes
Total vessels length	−4.63808	0.001774	Yes
Average vessels length	−3.00747	0.011889	Yes
Total number of end points	−5.73169	0.000612	Yes
			

**TABLE 2C:** Statistical comparisons VS vs. Control (*t*-test for 2 independent means).

**Parameter**	***t*-value**	***p*-value**	**Statistical significance**
			**at *p* < 0.05**

Vessels area	0.03348	0.487447	No
Vessels% area	1.48253	0.10617	No
Number of junctions	–0.09334	0.46506	No
Total vessels length	–2.81273	0.024092	Yes
Average vessels length	0.60302	0.289508	No
Total number of end points	–0.40821	0.352014	No

### TEM Processing

Macula utricles halves (specimens #3, #4, and #5 are from Meniere’s utricles and specimen #8 is from an VS utricle) were immersed in 4% paraformaldehyde, 2.5% glutaraldehyde for 1 day and then immersed in the following solutions: 2% OsO4 and 2% potassium ferricyanide (EMS, Fort Washington, PA, United States), 0.1% thio-carbohydrazide for 1 h, 2% OsO4 for 30 min, uranyl acetate 1% overnight, and 0.1% lead aspartate for 30 min (Ishiyama et al., 2017). Tissue was dehydrated in ascending ethyl alcohols and embedded in resin (Epon^®^, EMS). One-micron thick sections were made to identify the blood vessels at light microscopy, when the area of interest was visible, ultrathin 100 nm sections were obtained, and mounted on formvar coated single slot copper grids.

### TEM Qualitative Study

Systematic analysis was made in tissue sections containing the microvasculature in the stroma of the macula utricle. TEM observations and digital image capture were made using a FEI Tecnai transmission electron microscope T20 TEM -200 KV (Hillsboro, OR, United States). All sections are systematically analyzed at low (×3,500–5,000), and higher magnification view (×19,000–25,000). All sections were studied for the presence of vesicles in the VECs, pericytes, and perivascular BM alterations (i.e., thickening and disruption). TEM was used to identify ultrastructural changes, the distribution and alterations of tight junction morphology, abnormalities of cellular interactions between VEC and pericytes.

## Results

### Whole Mount Immunofluorescence

**FIGURE 2:**
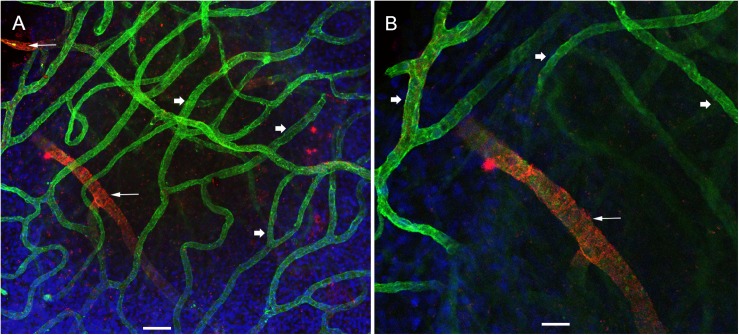
Whole mount preparation of the human macula utricle (VS) immunoreacted with antibodies against GLUT-1 and αSMA. **(A)** Shows a representative area of the macula utricle (low magnification view, 200×) from an VS patient (Specimen 6). Glucose transporter-1-IF (GLUT-1, green color, arrowheads), allowed to identify VECs in thin blood vessels (approximately 10–15 μm), αSMA-IF (red color) was seen in thick vessels (approximately 25 μm) were visualized (white thin arrows). DAPI stain cell nuclei (blue). **(B)** Is a high magnification view from **(A)**. Bar in **(A)** is 50 μm, in **(B)** is 25 μm.

Using immunofluorescence labeling and high-resolution laser confocal microscopy on whole mount preparations of the macula utricle obtained at surgery from patients diagnosed with MD and VS it was possible to identify VECs and pericytes of the microvascular network located in the stroma below the vestibular sensory epithelia. Figure 2A shows a representative area of the macula utricle (low magnification view) from one VS specimen. VECs were identified with antibodies against the glucose transporter-1 (GLUT-1). Uniform GLUT-1 labeling was observed in blood vessels (thick arrowheads). Three sizes of blood vessels were identified: Small size (<15 μm), medium (>15 to <25 μm) and large size (>25 μm).

αSMA-IF was seen in pericytes that surround in medium and large size vessels (>15 μm). The αSMA-IF pericytes in the large size vessels showed a protuberant soma and encircling circumferential processes (arrows in Figure 2A). The lack of αSMA-IF around GLUT-1 blood vessels of small size suggest that pericytes in these vessels express a different immunomarker as it has been seen in animal models (Shi et al., 2008). Figure 2B, shows the normal appearance of the blood vessels (high magnification view from Figure 2A).

### Pericytes and VECs Showed Marked Alterations in the Blood Vessels of Meniere’s Specimens

**FIGURE 3:**
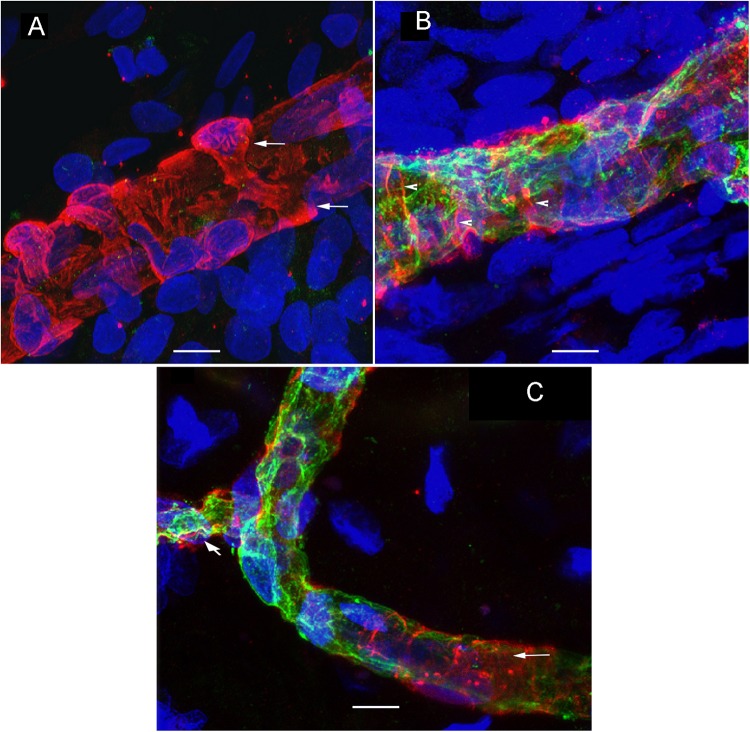
Double immunolabeling and high-resolution laser confocal microscopy we detected alterations in the macula utricle BLB. **(A)** Shows αSMA-IF (red) in thick vessels from an VS utricle (Specimen 6), small arrows point to a wrapping pericyte, IF is uniform through the blood vessel. **(B)** Shows a thick vessel from a Meniere’s patient (Specimen 1), there is disorganization of the pericyte processes (arrowheads) and there is evidence of degeneration of the VECs with an uneven lumen and the vessel wall exhibits areas of severe thinning (green color). **(C)** Shows constriction in the blood vessels (diagonal arrowhead). Small white arrow point to the blood vessel pericyte. DAPI stain cell nuclei (blue). Bar in **(A,B)** is 10 μm, in **(C)** is 15 μm.

Using double immunolabeling and high-resolution laser confocal microscopy we identified pericytes in a thick sized blood vessel from a VS patient in Figure 3A. The pericytes showed protuberant soma and encircling circumferential processes that nearly cover the vessel uniformly. In contrast, there were signs of damage in the pericytes and VECs of the macula utricle from two MD patients (Figures 3B,C). The pericytes in the Meniere’s specimen do not uniformly encircle the vessel and the pericyte processes are thinner and there are areas of the vessel unprotected by the pericyte processes. The pericyte processes appear to be disorganized and there were fewer pericyte soma present. Figure 3C shows an area of constriction within the blood vessel, and at this region there were few encircling pericytes, and the VECs were disorganized forming an uneven lumen of the vessel.

### Whole Mount Preparation Allows a Quantitative Analysis of Vascular Network

**FIGURE 4:**
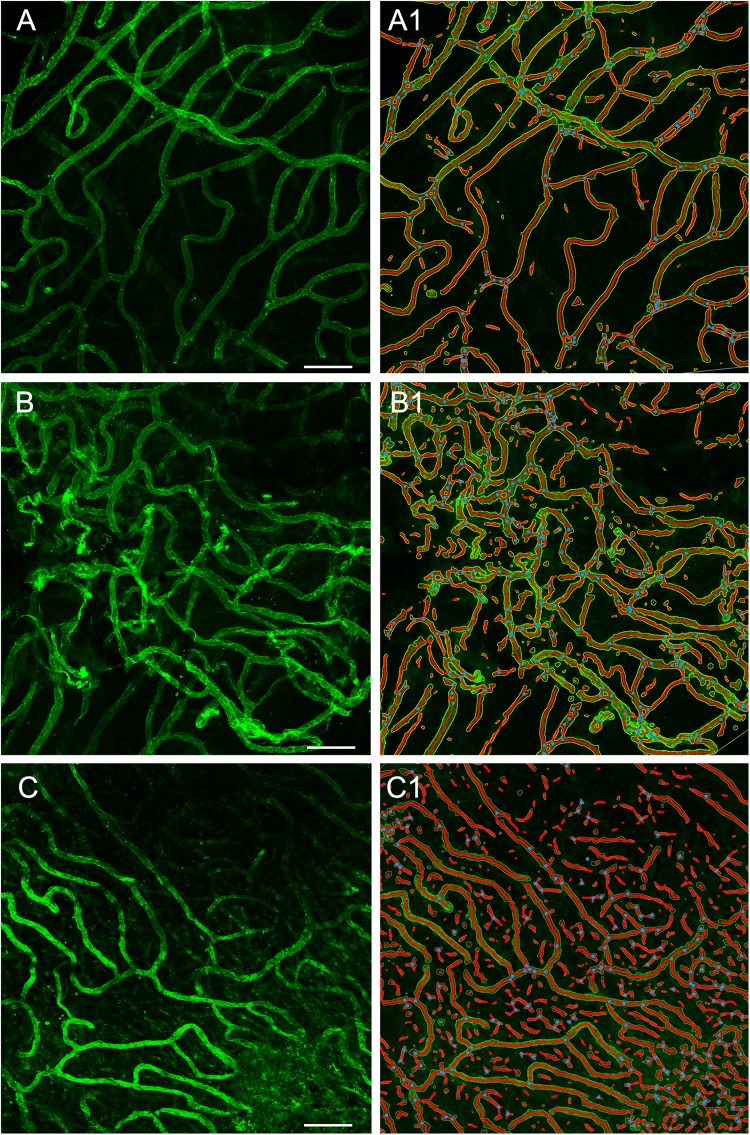
Whole mount preparation of the macula utricle allows a quantitative analysis of vascular network. Using GLUT-1-IF (green color) to delineate the vasculature it was possible to identify changes in the different specimens using AngioTool software. **(A–C)** Shows GLUT-1-IF in one VS utricle (Specimen 1), and two Meniere’s utricles (Specimen 2 and 3 respectively). **(A1–C1)** Shows the corresponding segmented images (From the left) created by the software. The segment images are then subjected to quantitative analysis (Table 1). Bar is 100 μm.

Using GLUT-1-IF to delineate the vasculature it was possible to identify and quantify changes in the different specimens. Figure 4, shows one VS and two MD specimens. Qualitative analysis showed a differential pattern of organization of the vasculature. The vasculature in Specimen 1 (VS) was well-organized, forming uniform patterns of branching and of size (Figure 4A). In contrast, in one of the Meniere’s utricle (Specimen 2), there were distorted vessels with an overall appearance of non-uniform bent and misshapen vessels, some shortened and twisted (Figure 4B). In contrast, there was a relatively normal appearance of blood vessels in specimen 3 (Figure 4C). Quantitative results are seen in Table 1. Figures 4A1–C1 shows the corresponding segmented images obtained with the software. Analysis of each image was achieved in less than 3 min, and the following the parameters were obtained: the vessels area, vessels percentage area, number of junctions, total vessels length, average vessel length and total number of endpoints (Table 1). Comparisons of the quantitative results were made as follow: MD vs. VS, MD vs. controls, and VS vs. controls. Tables 2A–C, shows that the number of junctions, total vessels length and average, and the number of endpoints were significantly smaller in MD specimens compared with VS specimens and controls. There were not statistical differences when we compared VS vs. controls, the only difference was in vessel length (Table 2C).

### Transmission Electron Microscopy

**FIGURE 5:**
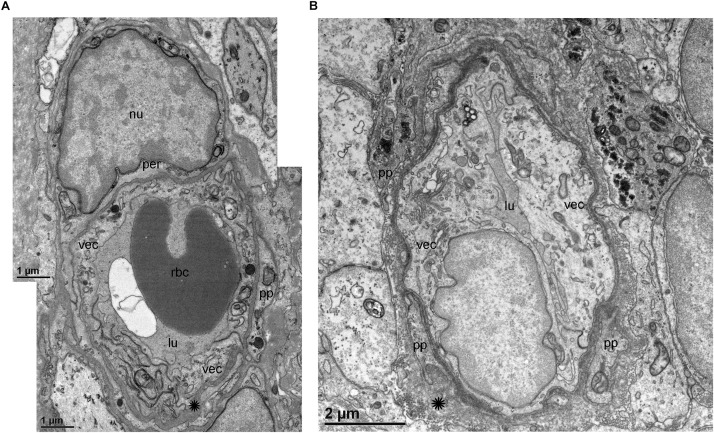
Transmission electron microscopy (TEM) of blood vessels from an VS and MD specimens. **(A)** Shows the normal organization of a blood vessel from an VS specimen. The lumen (lu) of the blood vessel is open and uniform, VECs (vec) showed no cytoplasmic vacuolization, and the pericytes (per) and processes (pp) are also normal, the perivascular basement membrane is compact with no vacuolization and compact (asterisk). **(B)** In contrast, the blood vessels of MD specimens showed consistently a narrow luminal space (lu) and the VECs (vec) showed vacuolization (thin arrow) and swollen cytoplasm, the pericytes processes (pp) showed also vacuolization, and the perivascular basement membrane shows disorganization, nu, cell nucleus; rbc, red blood cell. **(A)** Was obtained from specimen #8, and **(B)** from specimen #3. Thin sections (90 nm) were counterstained with uranyl acetate and lead nitrate.

**FIGURE 6:**
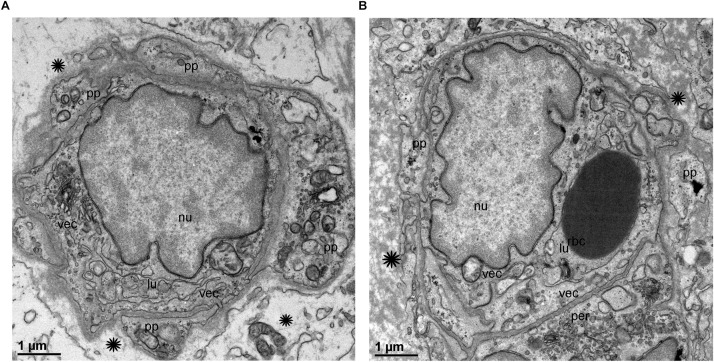
Transmission electron microscopy of blood vessels from MD specimens. **(A,B)** Showed a narrow luminal space (lu) and VECs (vec) showed swollen cytoplasm. The pericytes processes were vacuolated (thick arrowheads) **(A)**. The perivascular basement membrane is disorganized (asterisk). **(A)** Is from specimen #4, and **(B)** is from specimen #5 (respectively). Thin sections (90 nm) were counterstained with uranyl acetate and lead nitrate.

TEM analysis allowed the identification of changes in pericytes and VECs (Figures 5, 6). The blood vessels from the VS specimen showed an almost normal organization (Figure 5A, from Specimen #8). The lumen of the blood vessel is open and uniform, the VECs showed no cytoplasmic vacuolization and the pericytes are also normal. In the VS specimens, the perivascular basement membrane is well delineated and the stroma shows no signs of edema. In contrast the blood vessels of Meniere’s specimens showed consistently a narrow luminal space and the VECs showed vacuolization and swollen cytoplasm (Figures 5B, 6A,B), the pericytes processes showed also vacuolization. The perivascular basement membrane is disorganized and the vestibular stroma showed signs of edema. Figures 5B, 6A,B were obtained from Specimens #3, #4, and #5 respectively.

## Discussion

In the present study, we characterize and quantify the pathological changes in the microvasculature of patients diagnosed with MD. We have previously identified the cellular components of the BLB vasculature using antibodies against GLUT-1 and αSMA in formalin fixed cryostat sections of the utricular macula (Ishiyama et al., 2018). We were able to identify changes in the expression of these two proteins and other cellular markers (Ishiyama et al., 2018). However, it was not possible to identify differences in the vascular network as the analysis was limited to a select of sample of thin cross-sections of the utricle. For this reason, in the present study we used whole mount immunostained preparations to investigate changes in the microvasculature of the utricular macula in MD and compared with VS and control specimens.

There were similar changes in the expression of cellular markers and consistent ultrastructural alterations of the VECs, pericytes and perivascular basement membrane.

We also evaluated the degree of alteration in VECs and surrounding pericytes using TEM in these specimens. The ultrastructural changes correspond with the areas that our prior studies had demonstrated an upregulation of iNOS and the presence of nitrotyrosine, markers of oxidative stress (Ishiyama et al., 2017, 2018). In prior studies, we have detected downregulation of two basement membrane proteins in utricles of MD patients: collagen IV and laminin-beta, and upregulation of cochlin, the most abundant protein in the inner ear in the utricular stroma (Calzada et al., 2012). These results support the pathological leakage of the BLB in the vestibular endorgans from patients diagnosed with MD as a likely physiological mechanism causing vestibular and auditory dysfunction.

### Quantification of Alterations in the Vasculature

GLUT-1-IF was an excellent marker of VECs, that allow for the performance of automated assessment of the utricular macula vascular network. The use of this software allowed to determine that the number of junctions, total vessels length and average, and the number of endpoints were significantly smaller in Meniere’s specimens compared with VS and control specimens. An increase of the number of specimens for both groups would help to determine to what extent the vessels are affected in Meniere’s patients. These findings confirm the qualitative analysis of the visualization of the vasculature within the whole mounted specimens. The vasculature in the Meniere’s specimens exhibited disorganization, shortened and distorted vessels, quantified as a decrease in total vessel length, average vessel length. A significant decrease in the number of junctions and the total number of end points was found in MD specimens when compared with VS and control specimens. The information obtained with this preparation allowed comparison of the overall organization of the microvasculature, identify regional damages, atrophy and degree of vascularization. Quantitative changes using TEM will help to identify changes in discrete structures like tight junctions and vesicular transport.

### Mechanism for BLB Disruption in MD

Based on these results and our previous reports (Ishiyama et al., 2017, 2018) we suggest a possible mechanism for BLB disruption in MD and the subsequent signs of edema, that disrupt the homeostasis of the vestibular and auditory endorgans: (1) VECs exhibit signs of increased cellular permeability with increased transcytosis, possibly due to an ototoxic effect by an infection, noise or genetic predisposition. (2) Abnormally permeable VECs allow the transport of solutes to the abluminal portion of the capillary, sending signaling to the pericyte processes that are in close contact to the VECs (Ishiyama et al., 2017). (3) The morphological alterations and upregulation of oxidative stress marker iNOS and nitrotyrosine may be the pathophysiological change supporting the cellular damage of the VECs and pericytes (Ishiyama et al., 2018). MRI-FLAIR studies confirm the ipsilateral disruption of the BLB in MD to a much greater extent than that seen in sudden sensorineural hearing loss (Pakdaman et al., 2016). (4) The transport of solutes also affects the perivascular membranes which exhibit evidence of structural damage in the present study. (5) The leakage of fluids and ion homeostasis disruption affects the surrounding extracellular matrix which had been documented in our prior immunohistochemical studies of collagen IV in MD (Calzada et al., 2012). (6) These changes in the extracellular matrix, which is essential to protect and maintain the surrounding basement membrane, allows for abnormal thickening of the basement membrane noted in our early histopathological studies (McCall et al., 2009). (7) The alterations in the basement membranes of the vasculature may affect the transport of solutes from the basal to the apical portion of the supporting cells via disruption of the expression of aquaporins 4 and 6 (Ishiyama et al., 2010).

It is important to mention that the mechanisms of BLB dysfunction are likely far more complex, as other components of the BLB in the human remains to be investigated. The expression of tight junction proteins and the expression of proteins involved in transcellular transport have not yet been investigated as potential players in the abnormally high permeability of the vasculature in the BLB of MD, and these may reveal further target areas for treatment. Also, our study is focused on MD, however, the mechanism of damage to the BLB in other conditions such as noise induced hearing loss, exposure to ototoxic agents, and age related otopathologies remain to be investigated.

### Implications in Therapeutics

The delivery of local therapeutics to the inner ear is an active field for treating different types of disorders (Nyberg et al., 2019) and clinical trials begin to be implemented for different types of inner ear disorders. The challenge remains as there are several types of barriers in the cochlea and vestibular system (Rybak et al., 2019). Our results and previous studies in animal models on the normal organization of the utricle microvasculature shows that the organization of the BLB is quite similar, suggesting that the microvasculature could be used to deliver therapeutics into the inner ear. For the normal BLB, the delivery of therapeutics to the inner ear should be possible using the VECs transcellular transport mechanisms. As we have identified damage to the VECs it would be possible to use drugs that prevent or delay deterioration of these cells. Recent studies by Glueckert et al. (2015) proposed the use of nanoparticles in inner ear mouse explants. Nanoparticles could be applied systemically and VECs could internalize them via transcytosis. The knowledge of both normal and pathological organization of the microvasculature and the BLB is also relevant for local delivery because the inner ear blood supply is so intricate and varies in the different compartments.

The present study results demonstrate pathological changes in the microvasculature of the macula utricle from Meniere’s patients including disorganization of the vasculature, with abnormally shaped vessel lumen, uneven lumen with areas of constriction, abnormal branching and pericyte processes with decreased abluminal coverage and thinning, leaving the VECs exposed and unprotected. Quantitative analysis indicates that there are structural changes in the microvasculature of MD specimens, with a significantly decreased total vessel length and decreased average vessel length, and number of junctions and end points. This indicates that interventions aimed at preventing the damage to the microvasculature may help stop the progression of damage to the vestibular system, restoring balance and preventing vertigo spells. We believe that a similar process occurs in the cochlea of MD patients, and interventions aimed at preventing damage to the microvasculature may also help preserve hearing. The pathological changes found in the microvasculature of Meniere’s patients noted in this report and in our prior studies (Ishiyama et al., 2017, 2018) suggest that amelioration of vasoconstriction and BLB leakage may prevent chronic damage to vestibular endorgans. This may explain in part the temporary relief from vestibular symptoms when steroids are administered in MD.

### Limitations of This Study and Future Directions

Given the limited availability of human tissue specimens we did not perform quantitative immunohistochemistry to investigate changes with age, disease condition, or gender. There is also the need to identify VECs and pericytes in temporal bones from patients diagnosed with Meniere’s using celloidin embedded sections. The quantitative differences between specimens from patients with MD vs. VS must be interpreted with caution, given the small sample of patients analyzed, in addition VS vestibular endorgans are pathological specimens and thus are not true “normal” specimens, however, comparison with controls showed a similar vasculature organization.

Our study suggests that human inner ear tissue could be used to compare and contrast findings in animal models to design better therapies for vestibular and auditory disorders. The development of novel methods to identify and quantify blood vessels will allow the investigation of changes in the inner ear microvasculature in different animal models (Alves et al., 2018; Jiang et al., 2019) or pathological conditions.

## Conclusion

In the present study, we identified the microvasculature of patient’s diagnose with MD using whole mount preparations immunoreacted with antibodies against GLUT-1-IF and αSMA-IF. GLUT-1-IF was an excellent marker of VECs that allow to perform automated assessment of the macula utricle vascular network. αSMA-IF allowed to identify pericytes in large sized vessels, in contrast most of thin sized vessels were almost devoid of IF. The understanding of the complex intercellular communication between the VECs and pericytes would give directions to design potential drug therapies that alleviate and or prevent BLB disruption. The results allowed us to investigate the extent of intercellular disruption in the microvasculature of the macula utricle from Meniere’s patients. Our study suggest that human inner ear tissue could be used to compare and contrast findings in animal models to design better therapies for vestibular and auditory disorders.

## Data Availability Statement

The datasets generated for this study are available on request to the corresponding author.

## Ethics Statement

The studies involving human participants were reviewed and approved by the University of California, Los Angeles Institutional Review Board. The patients/participants provided their written informed consent to participate in this study.

## Author Contributions

GI designed the project, wrote the first draft of the manuscript, interpreted the histopathology and clinical data, and worked on the final manuscript. AI designed the project, collected the specimens at surgery, selected the patients, and reviewed the final manuscript. DA performed all the immunofluorescent staining and processed the tissue for TEM. IL developed all methodologies, collected all images, performed the quantitative analysis, wrote the results, edited the final manuscript, and coordinated all work related to the manuscript.

## Conflict of Interest

The authors declare that the research was conducted in the absence of any commercial or financial relationships that could be construed as a potential conflict of interest.

## Footnotes

1 http://angiotool.nci.nih.gov
